# Factors Affecting Patients Undergoing Cosmetic Surgery in Bushehr, Southern Iran

**Published:** 2012-07

**Authors:** Zeinab Salehahmadi, Seyyed Reza Rafie

**Affiliations:** Department of Plastic Surgery, Fatemeh Zahra Hospital, Bushehr University of Medical Sciences, Bushehr, Iran

**Keywords:** Cosmetic surgery, Factors, Iran

## Abstract

**BACKGROUND:**

Although, there have been extensive research on the motivations driving patient to undergo cosmetic procedures, there is still a big question mark on the persuasive factors which may lead individuals to undergo cosmetic surgery. The present study evaluated various factors affecting patients undergoing cosmetic surgery in Bushehr, Southern Iran.

**METHODS:**

From 24th March 2011 to 24th March 2012, eighty-one women and 20 men who wished to be operated in Fatemeh Zahra Hospital in Bushehr, Southern Iran and Pars Clinic, Iran were enrolled by a simple random sampling method. They all completed a questionnaire to consider reasons for cosmetic procedures. The collected data were statistically analyzed.

**RESULTS:**

Demographical, sociological and psychological factors such as age, gender, educational level, marital status, media, perceived risks, output quality, depression and self-improvement were determined as factors affecting tendency of individuals to undergo cosmetic surgery in this region. Trend to undergo cosmetic surgery was more prevalent in educational below bachelor degree, married subjects, women population of 30-45 years age group. Education level, age, marital status and gender were respectively the influential factors in deciding to undergo cosmetic surgery. Among the socio-psychological factors, self-improvement, finding a better job opportunity, rivalry, media, health status as well as depression were the most persuasive factors to encourage people to undergo cosmetic surgery too. Cost risk was not important for our samples in decision making to undergo cosmetic surgery.

**CONCLUSION:**

We need to fully understand the way in which the combination of demographic, social and psychological factors influence decision-making to undergo cosmetic surgery.

## INTRODUCTION

One of the most talked about forms of surgeries in the medical field is the result of a desire to feel satisfaction in one's own skin, the desire to be attractive, and the desire to feel happy with one's image. Cosmetic surgery history goes back to the early put of 20th century Eli’s lumpy ears amending. Shakespeare and Kellay (1997) defined cosmetic surgery as a kind of surgery to change the body’s appearance in the absence of any diseases, damages, born or con- genital deformation which can be a factor to improve individual’s life quality.^1^ Many cosmetic procedures were done to correct a disfig- urement due to an accident or birth defect. The majority of cosmetic surgery procedures done were for aesthetic reasons. Clearly, the most obvious benefit of a cosmetic procedure was considered an improvement in appearance. A better appearance would improve self-confidence and would provide a better body image. Though making the face and body prettier is not the only advantage of cosmetic procedure. Plastic surgery is a science and delicate art that helps a person to adapt himself or herself better and feel more self-confident. Plastic surgery certainly improves the psychological state of a person. Some studies have shown that plastic surgery operations may improve selfesteem and make people more sexually attractive. Thus, even small changes can about huge changes. Plastic surgery can be beneficial for both physical and mental health; however, it can also be hazardous if not performed correctly.^2^ The past decade witnessed an explosion in the number of cosmetic surgery procedures taking place in the World.^3^ It was shown that Iran is a country with the most frequency of undergoing plastic surgery, so it should be considered not only as a medical issue but also as a social matter.^4^ In today’s societies, some factors like emphasis on fashion, beauty as well as the presented images in cinema, magazines, media and so on have increased people’s motivation to undergo cosmetic surgery.^5^ On a global scale, the possible reasons for this increasing demand includes higher disposable incomes, advances in cosmetic surgery, loss of stigma, and the way in which cosmetic surgery is portrayed in the mass media and entertainment industries.^6^ Researchers have introduced such elements as sociocultural conditions, norms pressure, family, friends, media (satellite, magazine, internet, TV) as well as technology (medical systems and beauty industry) as the influential factors on people’s motivation to undergo cosmetic surgery.^7^ It has been demonstrated that the surge of television programs and articles, internet, and advertisements for cosmetic surgery has undoubtedly led to greater public awareness of cosmetic surgery.^8^ It was reported that a change in perceptions can be accounted primarily by increased media attention. It is also the case that many more people now personally know someone who has undergone cosmetic surgery, leading to a breakdown of previously held stereo-types and an increase in the number of people considering elective cosmetic surgery.^9^ It is said that within the psychological literature on cosmetic surgery, the focus until recently has been almost on the reasons for seeking cosmetic surgery and possible psychological consequences of such surgery.^10^ The reports revealed that the literature stems from cosmetic surgeons need to assess the psychological suitability of their patients for various procedures.^11^ Similarly some researchers believed that research on the factors influencing the future likelihood of having cosmetic surgery is sparse, and the available literature is typically focused on the body image of clinical or non-representative samples of women considering augmentative surgery.^12^ In one study, it was found that among a sample of females with undergraduate educational level, greater vicarious experience among friends and family who had done cosmetic surgery predicted greater likelihood of having cosmetic surgery in the future, most likely because it increased the amount of information that prospective patients had and because it broke down previously held stereotypes.^13^ Consistent with these suggestions, others also found that media exposure significantly predicted the likelihood of having cosmetic surgery.^13^ Reports revealed that women reported a greater likelihood of willingness to undergo cosmetic surgery than men, with older men the least likely to report such willingness. The sex bias in the willingness to undergo cosmetic surgery reflected with the likelihood of having cosmetic surgery.^14^ In a study in Iran (2011), it was shown that becoming more beautiful/handsome as well as rivalry were two main reasons for undergoing cosmetic surgery.^15^ In an attempt to understand the handling of cosmetic surgery in social science research, it was found that they chose three disciplines in which to explore cosmetic surgery; psychology, sociology, and history. In exploring psychology, there was a hope for better understanding the role of individuals’ thoughts and behaviors regarding cosmetic surgery, as well as any clinical findings related to this practice. The goal in studying sociology was to see how researchers addressed cosmetic surgery as a product of contemporary society and culture, and the relationship of the individual in society to this process. The aim in exploring history was to see how cosmetic surgery has been viewed through the ages, and how cosmetic surgery’s past was related to its present state in society. These three elements were effective on people’s tendency to undergo cosmetic sur- gery.^16^ Benefit of plastic surgery was shown to improve the appearance. A better appearance may improve once self confidence and provide a better body image but making once face and body prettier is not the only advantage of plas- tic surgery. Plastic surgery is a science and delicate art that helps a person to adapt himself or herself in the surrounding and feel more self confident. People have always felt that with the time, their inner self stays young but their outside may change. Whenever there is a need or desire to change or improve their outer appear- ance, people can now choose the procedures offered at plastic surgery clinics. Plastic surgery certainly improves the psychological state of a person. The procedure often results to an increase in self-esteem and confidence. As we make the changes to achieve a certain look that we desire, we become more confident with ourselves and become more comfortable in our interaction with others. This is a positive factor in our socialization process and may even improve our interaction skills. Correcting certain malformations in the face and body can help the person function better and more comfortably. These results may help the person live a happier life as worries about not being accepted by the society because of appearance may be eliminated. It may also increase an individual’s productivity. As improvements in appearances may increase chances of getting hired for a particular job, the person who has undergone plastic surgery may also increase his chances of becoming successful with career.^17^ It was noticed that operative factors on innovation adoption by help of TAM-TBP integrated model in which individuals attitudes were influenced by the perceived risk consisted of risks structures, social risk, time risk, financial risk along with security risk.^18^ In a research on the effective factors on cosmetic surgeries in Isfahan city in Iran; age, gender and marital status were introduced as effective factors on individuals trend to undergo different kinds of cosmetic surgeries.^19^ It was noticed that applicants of Botulinum Toxin injection in Tehran private clinics, Iran, depression was influential on individuals decision to undergo cosmetic actions.^20^ A research in Iran (2012) in Mahabad city showed that applicants for cosmetic surgery had some clinical psychiatric symptoms, liked depression and anxiety.^21^ This study evaluates factors affecting patients undergoing cosmetic surgery in Bushehr, Southern Iran.

## MATERIALS AND METHODS

Sample selection method was a simple random sampling one. 135 questionnaires were distributed in Fatemeh Zahra Hospital in Bushehr Province, Southern Iran as well as Pars Clinic patients who wished to undergo a cosmetic surgery. Data collection was done from March 2011 to March 2012. From these 135 questionnaires, 120 questionnaires were returned which 101 of them were valid for the study. Data collection method was a structured interview, document review and questionnaire. For ensuring that respondents did not have any problem to answer questions and as with the contents of questionnaire, pre-testing and pilottesting were essential before distributing the questionnaires. Pre-testing was done by consulting with some cosmetic surgeons and university professors. Some modifications and adjustments were made to the original questionnaire. For a better understanding, a pilot study was conducted among 20 patients. It assured us that the questionnaire was appropriate and the questions were generally understandable. The Cronbach’s alpha value was calculated for reliability of each factor in the questionnaire. Of 20 gathered questionnaires, all values were over-recommended level of 0.7. The final questionnaire consisted of two sections, first section had general questions consist of demographic questions which gathered demographic information such as gender, age, marital status and educational level of respondents. These questions were designed in multiple choice form and respondents can choose which was more applicable for them. The second part of questionnaire that was about cosmetic surgery was designed in 7- points Lickert scale. This kind of scaling included strongly disagree, disagree, almost disagree, no idea, almost agree, agree and strongly agree. Respondents can choose just one of the option from “1” strongly disagree to “7” strongly agree.

Reliability and validity were evaluated for reducing the possibility of getting incorrect answers in the research study. Validity and reliability in this research were done two times. The first time was done by the Expert Judgment for measuring the quality of the questionnaire, before the distribution. The second time was done after data collection. For that reason, Cronbach test was used for reliability and factor analysis for validity.

Reliability analysis allowed studying the properties of measurement scales and the items that make them increase. The reliability was tested two times by Cronbach alpha test. At first it was done on 20 questionnaires as a pilot test and mentioned in pilot test subsection. However, after collecting all data, the reliability of whole data was measured. So, the Cronbach alpha for each factor in the questionnaire was calculated. The reliability for all factors were more than recommended level of 0.7.

Validity was concerned whether the findings were really what they appeared to be. The first time, it was performed on 20 questionnaires which were mentioned on pilot test subsection. Measurement instrument validity was analyzed by factor analysis. In this study, each measurement criterion was considered as a distinct construct. The most common decisionmaking technique to obtain factors was to consider factors with Eigen value of over one as significant. According to factor analysis, percentage of total variance were over the recommended level of 50%.

In this project, SPSS software (Version 17.0, Chicago, IL, USA) was used for analyzing the collected data. Pearson Linear Correlation Coefficient, Spearman Correlation Coefficient, Regression analysis, Coefficient of Determination- Kruskal-Wallis tests were applied for analyzing the collecting data.

## RESULTS

**Figure1:**
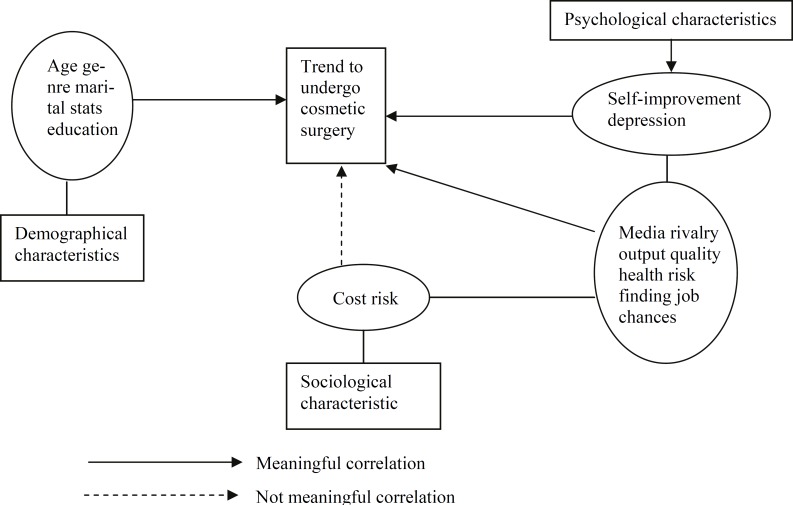
Research Effective Factors Model

According to Table 1, 20 (1.980%) of patients were male and 81 (80.19%) were female. Females had more tendency to undergo cosmetic surgery. More than 45 years old respondents were the minimum age group. However, the respondents between 30 and 45 years were the most cosmetic patients group, while 38 (37.62%) of them were less than 30 years old. 29 (28.71%) patients were single, while 72 (71.28%) were married**. **55 (54.45%) were in educational level less than bachelor degree, 23 (22.77%) had bachelor degree, 17 (16.83%) were in master educational level. Table 2 shows education level, age, marital status and gender were respectively the influential factors to decide to undergo cosmetic surgery. Based on Table 3 among the socio-psychological factors, self-improvement, finding a better job opportunity, rivalry, media, health status as well as depression were the most persuasive factors to encourage people undergoing cosmetic surgery too. Figure 1 summarizes factors, the effective and ineffective ones.

**Table 1:** Frequency and percentage of demographic characteristics

**Variable**		**No.**	**%**	**Total (%)**
Gender	Male	20	1.980	100
	Female	81	80.19	
Age	<30	38	37.62	100
	30-45	58	57.42	
	>45	4	3.96	
Marital status	Single	29	28.71	100
	Married	72	71..28	
Education level	<Bachelor	55	54.45	100
	Bachelor	23	22.77	
	Master	17	16.83	
	PhD or Dr	5	4.95	

**Table 2:** Kruskal-Wallis results of demographic features affecting on individual tendency to undergo cosmetic surgery.

**Variable**	**No.**	**Median**	**Av Rank**	**Z**
Gender				
Male	20	5.000	59.3	1.41
Female	81	5.000	49.5	-1.41
Age (years)				
30-45	59	5.000	51.8	0.33
<30	38	5.000	51.7	0.20
>45	4	3.500	32.1	-1.26
Marital status				
Single	29	5.000	45.1	-1.28
Married	72	5.000	53.4	1.28
Education level				
<Bachelor	17	5.000	52.0	0.15
Bachelor	23	5.000	44.7	-1.17
Master	56	5.000	53.0	0.77
PhD or Dr	5	6.000	54.2	0.25

**Table 3:** Analysis of socio-psychological characteristics affecting results on individuals tendency to undergo cosmetic surgeries.

**Variable**	**Trend to undergo cosmetic sur- gery**	**Media effect**
Correlation sig. 2 tailed	1	0/481
Trend to undergo cosmetic surgery	101	0/000
No.		101
Correlation sig. 2 tailed	0/481	1
media effect	0/000	101
No.	101 Trend to undergo cosmetic surgery	Cost Risk
Correlation sig. 2 tailed Trend to undergo cosmetic surgery	0.1oo 0.319	1
No.	101	101
Correlation sig. 2 tailed	0/100	1
cost risk	0/319	
No.	101	101
Correlation sig. 2 tailed	Trend to undergo cosmetic surgery 1	Health Risk 0/382
Trend to undergo cosmetic surgery	101	0/000
No.		101
Correlation sig. 2 tailed	0/38	1
health risk	0/000	101
No.	101 Trend to undergo cosmetic surgery	Rivalry
Correlation sig. 2 tailed	1	0/504
Trend to undergo cosmetic surgery	101	0/000
No.		101
Correlation sig. 2 tailed	0/504	1
rivalry	0/000	101
No.	101 Trend to undergo cosmetic surgery	Finding Better Romantic & Job
Correlation sig. 2 tailed	1	Opportunities 0/537
Trend to undergo cosmetic surgery	101	0/000
No.		101
Correlation sig. 2 tailed	0/537	1
Finding better romantic and job op-	0/000	101
portunities No.	101 Trend to undergo cosmetic surgery	Individuals Depression State
Correlation sig. 2 tailed	1	0/348
Trend to undergo cosmetic surgery	101	0/000
No.		101
Correlation sig. 2 tailed	0/348	1
Individuals depression state	0/000	101
No.	101 Trend to undergo cosmetic surgery	Individuals self-Improvement
Correlation sig. 2 tailed	1	0/547
Trend to undergo cosmetic surgery	101	0/000
No.		101
Correlation sig. 2 tailed	0/547	1
Individual self-improvement	0/000	101
No.	101	

## DISCUSSION

The results of this study highlighted a number of interesting findings and extended previous works on the likelihood of having cosmetic surgery in several ways. First of all, tendency to undergo a cosmetic surgery was shown to be affected by factors such as gender, age, marital status and education level. These results are in line with previous works.^19^ Second, the tendency to undergo cosmetic surgery was more prevalent in women population than men.^14^ This result which appears to be fairly robust can be traced back to the greater socio-cultural pressure on women than men to attain and incorporate ideals of physical and sexual attractiveness again which is in agreement with many others.

Our findings showed that most of our respondents were female, married and undergraduate. The 80% share of women in sample size demonstrates that beauty was of more importance for women in comparison to men. This may have origin in womanly union and beauty principle or women more dependency on fashion. However; this changed them to the^6^ factors affecting cosmetic surgery prevailing consumers of cosmetic surgery. The most of our respondents were married showing that married population were just as eager as single ones to undergo cosmetic surgery in order to gain more support of social, family, friends, partners along with acquiring more social positions and values and to be more attractive for others. As most of our respondents were in undergraduate level, we can conclude that sexual as well as environmental attraction caused them to undergo cosmetic surgery more. Third, sociological factors such as media exposure, perceived risks, rivalry, job chances were influential factors on our sample trend to undergo cosmetic surgery, and these were in line with previous studies too.^5^-^9^,^13^,^15^,^17^,^18^

Cost risk was not important for our samples in decision making for undergoing cosmetic surgery. Clearly, this particular association requires further research, which would benefit from the use of more sophisticated tools. Among the sociological factors, finding a better job as well as rivalry were the most important ones. As we make the changes to achieve a certain look that we desired, we became more confident with ourselves and became more comfortable in our interaction with others. This is a positive factor in our socialization process and may even improve our interaction skills. Correcting certain malformations in the face and body can help the person function better and more comfortably. These results may help a person have a happier life as worries about not being accepted by the society because of appearance that may be eliminated. It may also increase an individual’s productivity. As improvements in appearances may increase chances of getting hired for a particular job, the persons who had undergone plastic surgery might also increase their chances of becoming successful with the career. Fourth, psychological factors such as self-improvement, depression state were the influential factors on our sample tendency to undergo cosmetic surgery and these were in line with other researches.^10^,^11^,^16^,^20^,^21^

Among psychological factors as our findings showed self- improvement was the most effective factor on our population tendency to undergo cosmetic surgery. Self-improvement is essential to the nature of mankind. Because human beings have always sought selffulfillment through self-improvement, plastic surgery improvment and restoring form and function may be one of the world's oldest healing art for an increase in self-esteem and confidence. Although most researches have introduced the application of cosmetic surgery for depressed people, our findings showed that depression state was not too high. So those who decide to undergo cosmetic surgery, necessarily did not suffer from depression. To have a clear conclusion in this regard, a psychological test is recommended before doing any kind of cosmetic surgery. Future works could also extend the present findings by examining the association between the likelihood of having cosmetic surgery and such variables as cost.

In conclusion, the present study agrees with works documenting the important roles of demographic, social and psychological factors affecting individuals’ likelihood to undergo cosmetic surgery. Although discussions concerning the ultimate negative and positive effects of having cosmetic surgery continue unabated, it seems clear that the choice of having cosmetic surgery is influenced by social factors. Researchers would need to fully understand the way in which these factors combine to influence decision-making, as a focus on individual or social factors alone would likely result in an altogether one-sided account. The bottom line is that plastic surgery, no matter how minor, is still is a medical procedure that can affect an individual physical well-being and health. A little change might be sufficient enough to give an individual a big boost.
